# 1-[3-(4-Chloro­phen­yl)-5-(4-meth­oxy­phen­yl)-4,5-dihydro-1*H*-pyrazol-1-yl]butan-1-one

**DOI:** 10.1107/S1600536812009105

**Published:** 2012-03-07

**Authors:** Hoong-Kun Fun, Ching Kheng Quah, S. Samshuddin, B. Narayana, B. K. Sarojini

**Affiliations:** a X-ray Crystallography Unit, School of Physics, Universiti Sains Malaysia, 11800 USM, Penang, Malaysia; b Department of Studies in Chemistry, Mangalore University, Mangalagangotri 574 199, India; c Department of Chemistry, P. A. College of Engineering, Nadupadavu, Mangalore 574 153, India

## Abstract

In the title compound, C_20_H_21_ClN_2_O_2_, the benzene rings form dihedral angles of 6.35 (5) and 81.82 (5)° with the mean plane of the 4,5-dihydro-1*H*-pyrazole ring (r.m.s. deviation = 0.145 Å). This latter ring adopts an envelope conformation with the CH grouping as the flap. The dihedral angle between the benzene rings is 75.63 (4)°. In the crystal, mol­ecules are linked by C—H⋯Cl and C—H⋯O hydrogen bonds into chains along [-201]. The crystal structure also features C—H⋯π inter­actions.

## Related literature

For a related structure, see: Fun *et al.* (2010 ▶). For the stability of the temperature controller used in the data collection, see: Cosier & Glazer (1986 ▶). For standard bond lengths, see: Allen *et al.* (1987 ▶).
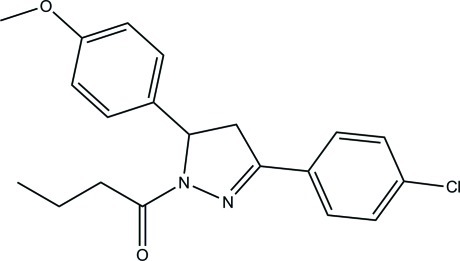


## Experimental

### 

#### Crystal data

C_20_H_21_ClN_2_O_2_
*M* *_r_* = 356.84
Triclinic, 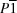

*a* = 6.7918 (3) Å
*b* = 10.8822 (4) Å
*c* = 13.2576 (5) Å
α = 109.202 (1)°
β = 91.396 (1)°
γ = 105.087 (1)°
*V* = 886.93 (6) Å^3^
*Z* = 2
Mo *K*α radiation
μ = 0.23 mm^−1^
*T* = 100 K
0.43 × 0.17 × 0.14 mm

#### Data collection

Bruker SMART APEXII DUO CCD diffractometer
Absorption correction: multi-scan (*SADABS*; Bruker, 2009 ▶) *T*_min_ = 0.908, *T*_max_ = 0.968
17324 measured reflections
6403 independent reflections
5492 reflections with *I* > 2σ(*I*)
*R*_int_ = 0.021

#### Refinement

*R*[*F* ^2^ > 2σ(*F* ^2^)] = 0.035
*wR*(*F* ^2^) = 0.104
*S* = 1.04
6403 reflections
228 parameters
H-atom parameters constrained
Δρ_max_ = 0.43 e Å^−3^
Δρ_min_ = −0.30 e Å^−3^

### 

Data collection: *APEX2* (Bruker, 2009 ▶); cell refinement: *SAINT* (Bruker, 2009 ▶); data reduction: *SAINT*; program(s) used to solve structure: *SHELXTL* (Sheldrick, 2008 ▶); program(s) used to refine structure: *SHELXTL*; molecular graphics: *SHELXTL*; software used to prepare material for publication: *SHELXTL* and *PLATON* (Spek, 2009 ▶).

## Supplementary Material

Crystal structure: contains datablock(s) global, I. DOI: 10.1107/S1600536812009105/hb6658sup1.cif

Structure factors: contains datablock(s) I. DOI: 10.1107/S1600536812009105/hb6658Isup2.hkl

Supplementary material file. DOI: 10.1107/S1600536812009105/hb6658Isup3.cml

Additional supplementary materials:  crystallographic information; 3D view; checkCIF report

## Figures and Tables

**Table 1:** Hydrogen-bond geometry (Å, °) *Cg*1 is the centroid of the C1–C6 benzene ring.

*D*—H⋯*A*	*D*—H	H⋯*A*	*D*⋯*A*	*D*—H⋯*A*
C11—H11*A*⋯Cl1^i^	0.93	2.73	3.4539 (10)	135
C14—H14*A*⋯O2^ii^	0.93	2.51	3.3253 (12)	147
C18—H18*A*⋯*Cg*1^iii^	0.97	2.62	3.4514 (9)	144

Symmetry codes: (i) 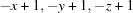; (ii) 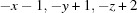; (iii) 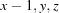.

**Fig. 1.**
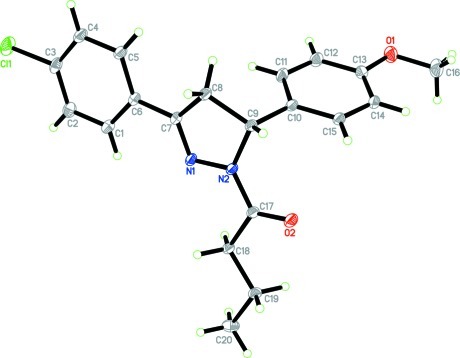
The molecular structure of the title compound showing 50% probability displacement ellipsoids for non-H atoms.

**Fig. 2.**
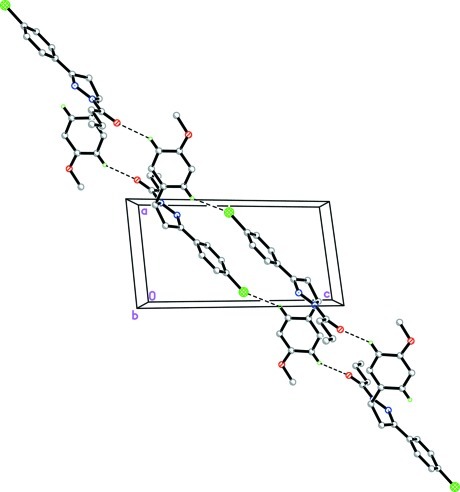
The crystal structure of the title compound, viewed along the b axis. H atoms not involved in hydrogen bonds (dashed lines) have been omitted for clarity.

## supplementary crystallographic information

### Comment

In continuation of our work on the synthesis and sturctures of pyrazoline derivatives (Fun *et al.*, 2010), the title compound (I) is prepared and its crystal structure is reported.

In the title molecule (Fig. 1), the two benzene rings (C1-C6 and C10-C15) form dihedral angles of 6.35 (5) and 81.82 (5)°, respectively, with the mean plane of 4,5-dihydro-1*H*-pyrazole ring (N1/N2/C7-C9, r.m.s. deviation = 0.145 Å). The dihedral angle between the two benzene rings is 75.63 (4)°. Bond lengths are comparable with a related structure (Fun *et al.*, 2010).

In the crystal structure, Fig. 2, molecules are linked *via* C11–H11A···Cl1 and C14–H14A···O2 hydrogen bonds (Table 1) into chains along [-201]. The crystal structure is further consolidated by C18–H18A···Cg1^iii^ (Table 1) interactions, where Cg1 is the centroid of C1-C6 benzene ring.

### Experimental

A mixture of (2*E*)-1-(4-chlorophenyl)-3-(4-methoxyphenyl)prop-2-en-1-one (2.72 g, 0.01 mol) and hydrazine hydrate (0.5 ml, 0.01 mol) in 25 ml butyric acid was refluxed for 8 h. The reaction mixture was cooled and poured into 50 ml ice-cold water. The precipitate was collected by filtration and purified by recrystallization from ethanol. Colourless blocks of (I) were grown from a DMF solution by slow evaporation and yield of the compound was 76% (*m.p.* : 369 K).

### Refinement

All H atoms were positioned geometrically and refined using a riding model with C–H = 0.93 or 0.98 Å and *U*_iso_(H) = 1.2 or 1.5 *U*_eq_(C). A rotating group model was applied to the methyl groups.

### Crystal data

**Table:**

C_20_H_21_ClN_2_O_2_	*Z* = 2
*M**_r_* = 356.84	*F*(000) = 376
Triclinic, *P*1	*D*_x_ = 1.336 Mg m^−^^3^
Hall symbol: -P 1	Mo *K*α radiation, λ = 0.71073 Å
*a* = 6.7918 (3) Å	Cell parameters from 8526 reflections
*b* = 10.8822 (4) Å	θ = 3.1–32.7°
*c* = 13.2576 (5) Å	µ = 0.23 mm^−^^1^
α = 109.202 (1)°	*T* = 100 K
β = 91.396 (1)°	Block, colourless
γ = 105.087 (1)°	0.43 × 0.17 × 0.14 mm
*V* = 886.93 (6) Å^3^	

### Data collection

**Table:**

Bruker SMART APEXII DUO CCD diffractometer	6403 independent reflections
Radiation source: fine-focus sealed tube	5492 reflections with *I* > 2σ(*I*)
Graphite monochromator	*R*_int_ = 0.021
φ and ω scans	θ_max_ = 32.7°, θ_min_ = 1.6°
Absorption correction: multi-scan (*SADABS*; Bruker, 2009)	*h* = −10→9
*T*_min_ = 0.908, *T*_max_ = 0.968	*k* = −16→16
17324 measured reflections	*l* = −20→20

### Refinement

**Table:**

Refinement on *F*^2^	Primary atom site location: structure-invariant direct methods
Least-squares matrix: full	Secondary atom site location: difference Fourier map
*R*[*F*^2^ > 2σ(*F*^2^)] = 0.035	Hydrogen site location: inferred from neighbouring sites
*wR*(*F*^2^) = 0.104	H-atom parameters constrained
*S* = 1.04	*w* = 1/[σ^2^(*F*_o_^2^) + (0.0556*P*)^2^ + 0.2279*P*] where *P* = (*F*_o_^2^ + 2*F*_c_^2^)/3
6403 reflections	(Δ/σ)_max_ = 0.001
228 parameters	Δρ_max_ = 0.43 e Å^−^^3^
0 restraints	Δρ_min_ = −0.30 e Å^−^^3^

### Special details

**Table:**

Experimental. The crystal was placed in the cold stream of an Oxford Cryosystems Cobra open-flow nitrogen cryostat (Cosier & Glazer, 1986) operating at 100.0 (1) K.
Geometry. All esds (except the esd in the dihedral angle between two l.s. planes) are estimated using the full covariance matrix. The cell esds are taken into account individually in the estimation of esds in distances, angles and torsion angles; correlations between esds in cell parameters are only used when they are defined by crystal symmetry. An approximate (isotropic) treatment of cell esds is used for estimating esds involving l.s. planes.
Refinement. Refinement of F^2^ against ALL reflections. The weighted R-factor wR and goodness of fit S are based on F^2^, conventional R-factors R are based on F, with F set to zero for negative F^2^. The threshold expression of F^2^ > 2sigma(F^2^) is used only for calculating R-factors(gt) etc. and is not relevant to the choice of reflections for refinement. R-factors based on F^2^ are statistically about twice as large as those based on F, and R- factors based on ALL data will be even larger.

### Fractional atomic coordinates and isotropic or equivalent isotropic displacement parameters (Å^2^)

**Table:**

	*x*	*y*	*z*	*U*_iso_*/*U*_eq_	
Cl1	0.89607 (4)	0.72819 (3)	0.48730 (2)	0.02770 (7)	
O1	−0.64280 (11)	0.12922 (7)	0.66911 (6)	0.02244 (14)	
O2	−0.24411 (10)	0.75836 (7)	0.97896 (5)	0.01753 (13)	
N1	0.11361 (11)	0.74489 (7)	0.79068 (6)	0.01296 (12)	
N2	−0.00821 (11)	0.71541 (7)	0.86691 (6)	0.01292 (12)	
C1	0.42153 (13)	0.77985 (9)	0.65141 (7)	0.01642 (15)	
H1A	0.3354	0.8358	0.6629	0.020*	
C2	0.57466 (14)	0.79372 (10)	0.58516 (7)	0.01864 (16)	
H2A	0.5930	0.8594	0.5530	0.022*	
C3	0.70038 (13)	0.70779 (10)	0.56759 (7)	0.01822 (16)	
C4	0.67516 (13)	0.60834 (9)	0.61348 (7)	0.01807 (16)	
H4A	0.7585	0.5505	0.5995	0.022*	
C5	0.52253 (13)	0.59623 (9)	0.68109 (7)	0.01569 (15)	
H5A	0.5051	0.5304	0.7130	0.019*	
C6	0.39554 (12)	0.68211 (8)	0.70129 (6)	0.01347 (14)	
C7	0.24252 (12)	0.67322 (8)	0.77666 (6)	0.01296 (14)	
C8	0.22791 (12)	0.59223 (8)	0.85087 (7)	0.01436 (14)	
H8A	0.3390	0.6342	0.9095	0.017*	
H8B	0.2293	0.4998	0.8123	0.017*	
C9	0.01915 (12)	0.59689 (8)	0.89181 (6)	0.01294 (14)	
H9A	0.0297	0.6161	0.9696	0.016*	
C10	−0.15764 (12)	0.47169 (8)	0.83447 (6)	0.01292 (14)	
C11	−0.15456 (14)	0.38856 (9)	0.72900 (7)	0.01689 (15)	
H11A	−0.0403	0.4090	0.6939	0.020*	
C12	−0.31873 (14)	0.27611 (9)	0.67577 (7)	0.01846 (16)	
H12A	−0.3135	0.2218	0.6057	0.022*	
C13	−0.49209 (13)	0.24412 (9)	0.72721 (7)	0.01629 (15)	
C14	−0.49940 (13)	0.32638 (9)	0.83207 (7)	0.01572 (15)	
H14A	−0.6146	0.3067	0.8668	0.019*	
C15	−0.33179 (13)	0.43875 (8)	0.88443 (7)	0.01423 (14)	
H15A	−0.3366	0.4930	0.9546	0.017*	
C16	−0.82621 (16)	0.09749 (11)	0.71737 (10)	0.0288 (2)	
H16A	−0.9211	0.0160	0.6690	0.043*	
H16B	−0.8873	0.1709	0.7324	0.043*	
H16C	−0.7935	0.0843	0.7832	0.043*	
C17	−0.14102 (12)	0.78735 (8)	0.91064 (6)	0.01265 (14)	
C18	−0.14900 (12)	0.90227 (8)	0.87211 (6)	0.01306 (14)	
H18A	−0.1800	0.8672	0.7944	0.016*	
H18B	−0.0151	0.9688	0.8905	0.016*	
C19	−0.30931 (13)	0.97135 (8)	0.92125 (7)	0.01505 (14)	
H19A	−0.2782	1.0069	0.9990	0.018*	
H19B	−0.4434	0.9050	0.9030	0.018*	
C20	−0.31538 (16)	1.08654 (9)	0.88125 (8)	0.02108 (17)	
H20A	−0.4138	1.1302	0.9160	0.032*	
H20B	−0.3541	1.0508	0.8048	0.032*	
H20C	−0.1820	1.1513	0.8977	0.032*	

### Atomic displacement parameters (Å^2^)

**Table:**

	*U*^11^	*U*^22^	*U*^33^	*U*^12^	*U*^13^	*U*^23^
Cl1	0.02295 (12)	0.03579 (14)	0.02385 (12)	0.00841 (9)	0.01502 (9)	0.00879 (9)
O1	0.0177 (3)	0.0192 (3)	0.0253 (3)	−0.0002 (2)	0.0032 (2)	0.0053 (3)
O2	0.0185 (3)	0.0192 (3)	0.0194 (3)	0.0091 (2)	0.0097 (2)	0.0092 (2)
N1	0.0110 (3)	0.0152 (3)	0.0140 (3)	0.0049 (2)	0.0047 (2)	0.0057 (2)
N2	0.0127 (3)	0.0142 (3)	0.0155 (3)	0.0067 (2)	0.0061 (2)	0.0074 (2)
C1	0.0142 (3)	0.0208 (4)	0.0165 (3)	0.0072 (3)	0.0039 (3)	0.0075 (3)
C2	0.0175 (4)	0.0238 (4)	0.0162 (4)	0.0058 (3)	0.0050 (3)	0.0088 (3)
C3	0.0136 (3)	0.0243 (4)	0.0136 (3)	0.0046 (3)	0.0052 (3)	0.0030 (3)
C4	0.0143 (4)	0.0214 (4)	0.0175 (4)	0.0078 (3)	0.0053 (3)	0.0031 (3)
C5	0.0133 (3)	0.0176 (3)	0.0163 (3)	0.0064 (3)	0.0041 (3)	0.0043 (3)
C6	0.0105 (3)	0.0168 (3)	0.0130 (3)	0.0049 (3)	0.0027 (2)	0.0042 (3)
C7	0.0103 (3)	0.0150 (3)	0.0140 (3)	0.0044 (3)	0.0025 (2)	0.0050 (3)
C8	0.0120 (3)	0.0169 (3)	0.0178 (3)	0.0069 (3)	0.0042 (3)	0.0083 (3)
C9	0.0130 (3)	0.0143 (3)	0.0144 (3)	0.0065 (3)	0.0041 (3)	0.0065 (3)
C10	0.0130 (3)	0.0142 (3)	0.0144 (3)	0.0062 (3)	0.0047 (3)	0.0066 (3)
C11	0.0159 (4)	0.0189 (4)	0.0159 (3)	0.0053 (3)	0.0071 (3)	0.0055 (3)
C12	0.0185 (4)	0.0191 (4)	0.0160 (3)	0.0048 (3)	0.0054 (3)	0.0038 (3)
C13	0.0151 (3)	0.0153 (3)	0.0192 (4)	0.0039 (3)	0.0028 (3)	0.0071 (3)
C14	0.0144 (3)	0.0170 (3)	0.0197 (4)	0.0060 (3)	0.0070 (3)	0.0098 (3)
C15	0.0157 (3)	0.0156 (3)	0.0150 (3)	0.0074 (3)	0.0061 (3)	0.0074 (3)
C16	0.0190 (4)	0.0264 (5)	0.0360 (5)	−0.0015 (4)	0.0059 (4)	0.0101 (4)
C17	0.0113 (3)	0.0130 (3)	0.0137 (3)	0.0050 (2)	0.0024 (2)	0.0035 (3)
C18	0.0122 (3)	0.0136 (3)	0.0150 (3)	0.0056 (3)	0.0033 (3)	0.0054 (3)
C19	0.0141 (3)	0.0148 (3)	0.0174 (3)	0.0073 (3)	0.0025 (3)	0.0045 (3)
C20	0.0252 (4)	0.0186 (4)	0.0230 (4)	0.0121 (3)	0.0016 (3)	0.0073 (3)

### Geometric parameters (Å, º)

**Table:**

Cl1—C3	1.7377 (9)	C9—H9A	0.9800
O1—C13	1.3661 (11)	C10—C15	1.3928 (11)
O1—C16	1.4285 (13)	C10—C11	1.3976 (11)
O2—C17	1.2305 (10)	C11—C12	1.3864 (12)
N1—C7	1.2952 (10)	C11—H11A	0.9300
N1—N2	1.3874 (9)	C12—C13	1.3981 (12)
N2—C17	1.3628 (10)	C12—H12A	0.9300
N2—C9	1.4862 (10)	C13—C14	1.3944 (12)
C1—C2	1.3878 (12)	C14—C15	1.3970 (12)
C1—C6	1.4036 (12)	C14—H14A	0.9300
C1—H1A	0.9300	C15—H15A	0.9300
C2—C3	1.3928 (13)	C16—H16A	0.9600
C2—H2A	0.9300	C16—H16B	0.9600
C3—C4	1.3829 (13)	C16—H16C	0.9600
C4—C5	1.3955 (12)	C17—C18	1.5106 (11)
C4—H4A	0.9300	C18—C19	1.5210 (11)
C5—C6	1.3983 (11)	C18—H18A	0.9700
C5—H5A	0.9300	C18—H18B	0.9700
C6—C7	1.4662 (11)	C19—C20	1.5222 (12)
C7—C8	1.5114 (11)	C19—H19A	0.9700
C8—C9	1.5376 (11)	C19—H19B	0.9700
C8—H8A	0.9700	C20—H20A	0.9600
C8—H8B	0.9700	C20—H20B	0.9600
C9—C10	1.5161 (11)	C20—H20C	0.9600
			
C13—O1—C16	116.96 (8)	C12—C11—H11A	119.4
C7—N1—N2	107.57 (7)	C10—C11—H11A	119.4
C17—N2—N1	122.26 (7)	C11—C12—C13	120.15 (8)
C17—N2—C9	124.98 (7)	C11—C12—H12A	119.9
N1—N2—C9	112.74 (6)	C13—C12—H12A	119.9
C2—C1—C6	120.57 (8)	O1—C13—C14	124.71 (8)
C2—C1—H1A	119.7	O1—C13—C12	115.63 (8)
C6—C1—H1A	119.7	C14—C13—C12	119.63 (8)
C1—C2—C3	118.99 (8)	C13—C14—C15	119.28 (8)
C1—C2—H2A	120.5	C13—C14—H14A	120.4
C3—C2—H2A	120.5	C15—C14—H14A	120.4
C4—C3—C2	121.74 (8)	C10—C15—C14	121.80 (8)
C4—C3—Cl1	119.37 (7)	C10—C15—H15A	119.1
C2—C3—Cl1	118.87 (7)	C14—C15—H15A	119.1
C3—C4—C5	118.89 (8)	O1—C16—H16A	109.5
C3—C4—H4A	120.6	O1—C16—H16B	109.5
C5—C4—H4A	120.6	H16A—C16—H16B	109.5
C4—C5—C6	120.66 (8)	O1—C16—H16C	109.5
C4—C5—H5A	119.7	H16A—C16—H16C	109.5
C6—C5—H5A	119.7	H16B—C16—H16C	109.5
C5—C6—C1	119.12 (8)	O2—C17—N2	119.86 (7)
C5—C6—C7	120.28 (7)	O2—C17—C18	123.61 (7)
C1—C6—C7	120.57 (7)	N2—C17—C18	116.52 (7)
N1—C7—C6	121.23 (7)	C17—C18—C19	112.44 (7)
N1—C7—C8	113.34 (7)	C17—C18—H18A	109.1
C6—C7—C8	125.25 (7)	C19—C18—H18A	109.1
C7—C8—C9	101.98 (6)	C17—C18—H18B	109.1
C7—C8—H8A	111.4	C19—C18—H18B	109.1
C9—C8—H8A	111.4	H18A—C18—H18B	107.8
C7—C8—H8B	111.4	C18—C19—C20	111.65 (7)
C9—C8—H8B	111.4	C18—C19—H19A	109.3
H8A—C8—H8B	109.2	C20—C19—H19A	109.3
N2—C9—C10	110.38 (6)	C18—C19—H19B	109.3
N2—C9—C8	99.94 (6)	C20—C19—H19B	109.3
C10—C9—C8	114.84 (7)	H19A—C19—H19B	108.0
N2—C9—H9A	110.4	C19—C20—H20A	109.5
C10—C9—H9A	110.4	C19—C20—H20B	109.5
C8—C9—H9A	110.4	H20A—C20—H20B	109.5
C15—C10—C11	117.92 (8)	C19—C20—H20C	109.5
C15—C10—C9	120.71 (7)	H20A—C20—H20C	109.5
C11—C10—C9	121.33 (7)	H20B—C20—H20C	109.5
C12—C11—C10	121.22 (8)		
			
C7—N1—N2—C17	170.91 (8)	C7—C8—C9—C10	98.74 (7)
C7—N1—N2—C9	−10.65 (9)	N2—C9—C10—C15	−91.37 (9)
C6—C1—C2—C3	0.94 (13)	C8—C9—C10—C15	156.62 (7)
C1—C2—C3—C4	0.68 (14)	N2—C9—C10—C11	86.38 (9)
C1—C2—C3—Cl1	−178.41 (7)	C8—C9—C10—C11	−25.64 (11)
C2—C3—C4—C5	−1.50 (14)	C15—C10—C11—C12	−0.50 (13)
Cl1—C3—C4—C5	177.60 (7)	C9—C10—C11—C12	−178.30 (8)
C3—C4—C5—C6	0.70 (13)	C10—C11—C12—C13	0.25 (14)
C4—C5—C6—C1	0.87 (13)	C16—O1—C13—C14	4.58 (13)
C4—C5—C6—C7	−176.92 (8)	C16—O1—C13—C12	−176.96 (8)
C2—C1—C6—C5	−1.70 (13)	C11—C12—C13—O1	−178.16 (8)
C2—C1—C6—C7	176.08 (8)	C11—C12—C13—C14	0.38 (13)
N2—N1—C7—C6	−179.36 (7)	O1—C13—C14—C15	177.66 (8)
N2—N1—C7—C8	−4.00 (9)	C12—C13—C14—C15	−0.74 (13)
C5—C6—C7—N1	−174.99 (8)	C11—C10—C15—C14	0.12 (12)
C1—C6—C7—N1	7.25 (12)	C9—C10—C15—C14	177.94 (7)
C5—C6—C7—C8	10.22 (12)	C13—C14—C15—C10	0.49 (12)
C1—C6—C7—C8	−167.54 (8)	N1—N2—C17—O2	−179.14 (7)
N1—C7—C8—C9	15.87 (9)	C9—N2—C17—O2	2.62 (12)
C6—C7—C8—C9	−168.98 (7)	N1—N2—C17—C18	−0.20 (11)
C17—N2—C9—C10	76.57 (10)	C9—N2—C17—C18	−178.45 (7)
N1—N2—C9—C10	−101.82 (8)	O2—C17—C18—C19	−4.07 (11)
C17—N2—C9—C8	−162.10 (8)	N2—C17—C18—C19	177.04 (7)
N1—N2—C9—C8	19.52 (8)	C17—C18—C19—C20	−179.85 (7)
C7—C8—C9—N2	−19.34 (8)		

### Hydrogen-bond geometry (Å, º)

Cg1 is the centroid of the C1–C6 benzene ring.

**Table:**

*D*—H···*A*	*D*—H	H···*A*	*D*···*A*	*D*—H···*A*
C11—H11*A*···Cl1^i^	0.93	2.73	3.4539 (10)	135
C14—H14*A*···O2^ii^	0.93	2.51	3.3253 (12)	147
C18—H18*A*···*Cg*1^iii^	0.97	2.62	3.4514 (9)	144

Symmetry codes: (i) −*x*+1, −*y*+1, −*z*+1; (ii) −*x*−1, −*y*+1, −*z*+2; (iii) *x*−1, *y*, *z*.
